# Acceptability and Feasibility of a Virtual Family-Based Single-Session Intervention for Young Teens at High Risk for Substance Use Disorder: Single-Arm Pilot Trial

**DOI:** 10.2196/93263

**Published:** 2026-09-29

**Authors:** Chelsea K Pike, Catherine Stanger, Inbal Nahum-Shani, Susan A Murphy, Vanessa Ramirez, Madison Siegel, Lucia Berglund, Amy Hughes Lansing

**Affiliations:** 1 Department of Psychological Science University of Vermont Burlington, VT United States; 2 Center for Technology and Behavioral Health Geisel School of Medicine Dartmouth College Lebanon, NH United States; 3 Institute for Social Research University of Michigan Ann Arbor, MI United States; 4 Departments of Statistics and Computer Science Harvard University Cambridge, MA United States

**Keywords:** internet-delivered intervention, digital health, single-session intervention, substance use, adolescents, parents

## Abstract

**Background:**

Preventive interventions to delay substance use disorder (SUD) are ideally delivered during early adolescence, a critical period for exposure to substance use (SU) risk factors. Young teens (aged 12-14 years) engaging in occasional use of alcohol, cannabis, or tobacco use, or with a family history of SUD, are at increased risk for SUD. Although behavioral-ecological family therapy is effective for prevention, many families face access barriers. Single-session interventions (SSIs) may offer an accessible, low-intensity alternative.

**Objective:**

This open pilot trial aimed to evaluate the acceptability, feasibility, and potential usefulness of a family-based SSI targeting improvements in family practices that prevent SUD.

**Methods:**

Participants were 9 young teens engaging in occasional SU or with a family history of SUD and their parent. The SSI involved a virtual 2-hour family consultation with a trained family coach. Acceptability was measured via Program Feedback Scale, feasibility via SSI completion rate, and potential usefulness via pre-post changes in parenting self-efficacy.

**Results:**

The SSI was acceptable to parents and teens (mean Program Feedback Scale scores ≥3) and feasible (8/9, 88.89% of parent-teen dyads completed the SSI). Among the 8 completing dyads, most participants (13/16, 81.25%) selected Communication and Problem Solving as their primary target for change. Parents reported increases in parenting self-efficacy from pre- to post-SSI.

**Conclusions:**

Findings support the SSI’s acceptability and feasibility for young teens at high risk for SUD. A randomized clinical trial is needed to confirm the SSI’s acceptability, feasibility, and efficacy, as well as to address potential for delivery by a lay workforce.

**Trial Registration:**

ClinicalTrials.gov NCT06538922; https://clinicaltrials.gov/study/NCT06538922

## Introduction

Preventive interventions that aim to delay the escalation of substance use (SU) and the onset of a substance use disorder (SUD) in adolescence might be best delivered during early adolescence, which is a critical developmental period for exposure to risk factors for SU [1-3]. Young teens (ie, aged between 12 and 14 years) in need of indicated preventive interventions include those occasionally engaging in SU and those having immediate family members with an SUD history. For example, young teens who occasionally use alcohol, cannabis, or tobacco before 15 years of age are at least 4-6 times more likely to experience an SUD [1-6]. Similarly, youth with a family history of SUD are 2-8 times more likely to experience an SUD [7-10]. SUDs are associated with substantial burden, accounting for over 13,000 deaths and 4.4 million disability-adjusted life years globally in 2021 [11]. Given these risks, there is a great need for effective and accessible indicated preventive interventions for young teens with early danger signs foreshadowing the development of an SUD.

The best practice indicated preventive intervention for SUD in young teens at high risk for SUD is behavioral-ecological family therapy [12-14]. These evidence-based indicated prevention programs help increase parents’ and teens’ capability, opportunity, and motivation to use family practices known to reduce risk for escalation of SU, such as increasing parental monitoring and teen time in prosocial activities, and supporting parent-teen relationship building. Behavioral-ecological family therapy programs shape family practices and relationship quality via extensive supported instruction and practice that addresses the family’s specific needs and social-ecological context. Although innovative approaches of embedding indicated prevention in school-based multilevel prevention programs have improved access to such programs [15-17], most families of young teens at high risk for SUD are unable to readily access family-based indicated prevention programs [18]. For families that do have access, there are often challenges with engaging parents and teens in starting and completing the programs [19], which typically consist of at least 10 weeks of treatment [20]. More accessible and acceptable family-based indicated prevention programs for young teens at high risk for SUD are needed to improve outcomes.

Single-session interventions (SSIs) show substantial promise as a method for delivering efficacious and accessible family-based interventions for young teens at high risk for SUD. SSIs are defined as “specific, structured programs that intentionally involve just one visit or encounter with a clinic, provider, or program” [21]. Importantly, multiple SSIs for parents and youth have been shown to be acceptable and effective in improving psychosocial outcomes. For example, SSIs for youth presenting to a mental health service and their families have been linked to significant improvements in youth psychopathology [22] and functioning [23] 1 month following the SSI, as well as decreases in both youth and parent worries about the youth’s mental health problem [24]. Additionally, youth and their families generally reported high levels of satisfaction with these interventions [22-24]. SSIs have also been shown to benefit family dynamics. For example, an SSI designed to help youth and their families develop a collaborative plan for managing youth mental health led to improvements in family communication [25].

Beyond targeting general mental health in the family context, SSIs and low-intensity interventions, those consisting of 4-5 sessions or fewer [26,27], have shown promise specifically for reducing SU in teens. For example, a meta-analysis of interventions consisting of 1-2 sessions found that they were effective in reducing teen heavy alcohol use and overall substance-related problems [28]. Similarly, a meta-analysis of SU interventions consisting of 1-4 sessions found that they reduced the frequency and quantity of teen alcohol consumption [29]. Although SSIs and low-intensity interventions appear to be effective and acceptable for behavior change in teens and families, there is a need for the development of SSIs for indicated family-based SUD prevention for young teens. These SSIs would need to flexibly target the full repertoire of family management and relationship skills involved in SU prevention in young teens at high risk for an SUD, allowing for the individualized assessment and intervention requisite in indicated prevention.

To meet these needs, the research team developed and tested a teen and parent SSI, with young teens at high risk for SUD and their parent. The primary aim of this study was to examine the acceptability and feasibility of this SSI. The potential usefulness of the SSI was also explored. Specifically, the study sought to determine whether (1) the SSI was acceptable to both parents and teens, (2) the SSI demonstrated adequate feasibility in terms of session completion, (3) parents and teens selected areas of challenge that aligned with the family challenges they reported, and (4) parents reported increased parenting self-efficacy following the SSI. It was expected that the SSI would be acceptable and feasible, that parents and teens could self-select areas of challenge, and that parents would report increased self-efficacy for family management skills for SUD prevention after completing the SSI.

## Methods

### Overview

For this open pilot trial, we tested the feasibility, acceptability, and potential usefulness of the SSI among 9 young teens at risk for SUD and their parents. Participants were recruited nationally through web-based advertisements. The study was preregistered at ClinicalTrials.gov (NCT06538922). Final sample size was determined by resource availability within the funding period.

### Participants

Inclusion criteria for the teen participants were (1) age 12-14 years, and (2) at least one of the following risk factors—(i) occasional SU per teen report on the Screening to Brief Intervention (S2BI [30]) initial screening questions (ie, teen reported use of alcohol, tobacco, or marijuana once or twice in the past year but not monthly or more), (ii) history of SUD in the immediate family, or (iii) current or past-year SU in a caregiver or sibling that lives with the teen per parent report. Additionally, both the teen and the parent participants were required to (3) be able to complete study activities in English and (4) have access to the internet. Exclusion criteria were (1) known diagnosis of SUD in the teen per parent report; (2) teen use of alcohol, tobacco, or marijuana monthly or more in the past year per teen report on the S2BI; (3) teen status as ward of the state; (4) active psychosis in the parent or teen; or (5) severe medical or psychiatric illness in the parent or teen that would prevent participation.

### Procedure

The recruitment goal for this pilot trial was up to 15 parent-teen dyads, with the final enrollment amount of 9 dyads determined by resource availability during the grant period. Dyads were recruited throughout the United States for 8 weeks between July 15, 2024, and September 10, 2024, using TrialFacts, a recruitment company that posted online advertisements directed at parents with concerns about their teen engaging in occasional SU. Advertisements were posted on a variety of platforms (eg, Facebook, Instagram, YouTube, Google, Yahoo, Bing, Reddit, and Quora) as determined by TrialFacts’ proprietary algorithm, with the majority of advertisements posted on Facebook, and included a prompt for families to complete an initial interest form and schedule a screening call. Research staff spoke with the parent and teen separately during the screening call to determine whether they met inclusion and exclusion criteria. Teens answered the S2BI items only after providing additional verbal consent and confirming that they were in a private space. Although their answers to the S2BI questions were spoken aloud, answer options did not reference SU to ensure teen confidentiality. All other eligibility criteria were assessed via parent report. All families, regardless of eligibility, were given resources for mental health and SU treatment and prevention. Families that met all eligibility criteria in the screening call were scheduled for their virtual SSI visit. Before and immediately after the SSI visit, parents and teens completed self-report surveys (see Measures section).

### Single-Session Intervention

#### Overview

The SSI consisted of a virtual 2-hour family consultation session conducted via Zoom (Zoom Communications, Inc) by a family coach. The primary family coach for this study was a staff member with a master’s degree in nutrition and food science and without any education in counseling or mental health, including none in nutrition counseling before this study. This family coach led 7 of the 9 sessions. A backup family coach with a master’s degree in clinical psychology led 2 sessions when the primary coach was not available. Both family coaches received the same training as part of this study. Training included 9 hours of didactics in the Everyday Parenting behavioral family therapy model [31], Collaborative Problem-Solving [32], and single-session consultations grounded in Solution-Focused Brief Therapy [33], as well as ongoing weekly supervision of SSI work by a licensed clinical psychologist. The primary aim of the SSI was to provide brief individualized family management skills training, and the approach was directly modeled on the single-session consultation method developed by Schleider et al [33]. The SSI began with the family coach providing the parent and teen with an overview of the single-session rationale and structure. The SSI was then delivered in 3 components, beginning with the teen (approximately 40 minutes), then the parent (approximately 55 minutes), and then the parent and teen together (approximately 20 minutes).

#### Teen-Only and Parent-Only Portions

Both the teen-only and parent-only portions of the SSI began with the family coach reviewing core family management skills: Communication and Problem Solving, Information Sharing, and, for parents only, Proactive Planning. Communication and Problem Solving emphasizes the importance of parents and teens connecting daily about small, routine topics, and working collaboratively to solve problems by listening to one another’s perspectives and developing mutually acceptable solutions. Information Sharing highlights the need for parental monitoring, including reciprocal communication by parents and teens about their daily plans, experiences, and whereabouts when apart from each other. Proactive Planning emphasizes the importance of parents communicating clear expectations for their teen’s behavior and activities as well as planning to ensure that their teen spends time under adult supervision and with peers engaging in healthy behaviors.

Following the skills overview, the family coach separately helped parents and teens select a top area of challenge in completing family management skills within the parent-teen relationship. Then, the family coach guided parents and teens in developing an individualized action plan consisting of 3 specific actions they could take to begin addressing the challenge over the next week. Action plan steps were ideally generated by the teen and the parent based on strategies that have worked in the past, with coaches offering evidence-based ideas for action plan steps when needed. Parents and teens also identified other individuals who could support them in following through on their action plans, as well as obstacles that might stand in their way and strategies for overcoming them. Finally, the family coach expressed confidence in the parent’s and teen’s ability to successfully implement the plan.

#### Joint Parent and Teen Portion

The final segment of the SSI brought the parent and teen back together. The family coach led the dyad through a structured sharing of challenges they had each identified and their corresponding action plans while modeling providing validation, asking questions, and offering supportive feedback about both action plans. The SSI ended with the teen and the parent setting a goal to try each of their action plans over the next week.

#### Coaching Fidelity

During each session, the family coach completed a fidelity checklist and documented any deviations from the planned session structure. Fidelity checklists were reviewed during a weekly supervision meeting with the study’s principal investigator (AHL). Additionally, each coach’s first session was supervised live by the principal investigator to confirm fidelity and readiness to deliver with independence.

### Measures

#### Substance Use

Parents reported whether they had ever had challenges with problematic SU or an SUD and also indicated whether they currently use, previously used, or had never used tobacco and cannabis [34,35]. Teen participants completed SU questions adapted from the 2023 Middle School Youth Risk Behavior Survey [36]. The questions assessed whether teens have ever used (yes or no) cigarettes, electronic vapor products, other tobacco products (eg, chewing tobacco and cigars), alcohol, and marijuana.

#### Mental Health

Teen mental health was assessed using the 17-item Pediatric Symptom Checklist (PSC-17 [37]), a parent report measure of youth psychosocial functioning. The PSC-17 includes 3 subscales: internalizing problems, externalizing problems, and attention problems. Parents rate the frequency of their child’s symptoms on a 3-point scale: 0 (never), 1 (sometimes), or 2 (often). Subscale scores are calculated by summing the relevant items, with scores of 5 or higher on the internalizing problems subscale and scores of 7 or higher on the externalizing and attention subscales indicating a positive screening in that domain. The PSC-17 has demonstrated validity in adolescents [38].

### Intervention Acceptability: Program Feedback Scale

Acceptability of the SSI was assessed using the Program Feedback Scale (PFS [39]), which was completed by both parents and teens immediately following the SSI. The 7-item self-report measure asks participants to indicate the extent to which they enjoyed, understood, found easy to complete, tried their hardest during, found helpful, would recommend, and agreed with what they learned in the SSI. Participants rate each item on a Likert scale from 1 (really disagree) to 5 (totally agree). Mean scores were calculated for the overall scale and for individual items, with higher mean scores indicating greater acceptability. The a priori criterion for acceptability specified that any mean scores below 3 would indicate a need for revision of the SSI (as in Schleider et al [40]). The PFS has been used to evaluate the acceptability of SSIs in prior studies [40-42].

### Intervention Feasibility: SSI Completion Rate

Feasibility of the SSI was assessed by examining the percentage of parent-teen dyads that completed the full SSI. The a priori criterion for feasibility specified that a completion rate below 80% would indicate a need for revision of the SSI.

### Intervention Potential Usefulness

#### Congruence of Participant-Selected Area of Challenge With Self-Reported Pre-SSI Family Functioning

Parent and teen responses to pre-SSI family functioning surveys were examined to determine whether parents and teens selected an area of challenge with family management skills during the SSI that aligned with the family challenges they reported in these measures.

#### Participant-Selected Area of Challenge

At the start of the SSI, each parent and teen separately selected 1 of 3 areas of challenge to work on during the session: Communication and Problem Solving, Information Sharing, or, for parents only, Proactive Planning. Prior to the SSI, parent-teen Communication and Problem Solving ability was assessed using the communication subscale of the Inventory of Parent and Peer Attachment (IPPA); parent-teen Information Sharing ability was assessed using the parental solicitation and child disclosure subscales of the Parental Monitoring Short Scale (PMSS), as well as the Parental Knowledge questionnaire; and parent-teen Proactive Planning ability was assessed using the parental control subscale of the PMSS.

#### Pre-SSI Family Functioning Surveys

Prior to the SSI, parents and teens each completed 5 items from the IPPA communication subscale [43]. Each item assessed the quality of teen communication with parent on a Likert scale from 1 (never true) to 5 (always true). Ratings were averaged across the 5 items, with higher scores indicating better communication. This 5-item version of the IPPA communication subscale has shown adequate reliability and validity in young teens [44,45]. In the present sample, the parent IPPA communication subscale demonstrated poor internal consistency (α=.59), while the teen subscale demonstrated acceptable internal consistency (α=.78).

Prior to the SSI, parents and teens each completed the PMSS, a 9-item self-report measure of parental monitoring. The PMSS has 3 subscales: parental control, parental solicitation, and child disclosure. Participants rate each item on a Likert scale from 1 (never) to 5 (always). Mean scores were calculated for each subscale, with higher scores indicating greater parental control, parental solicitation, and child disclosure. The PMSS has shown predictive validity in an adolescent sample [46]. Among parents in the present sample, the parental control subscale demonstrated good internal consistency (α=.83), and the parental solicitation (α=.63) and child disclosure (α=.66) subscales demonstrated acceptable internal consistency. Among teens in the present sample, the parental control subscale demonstrated poor internal consistency (α=.45), while the parental solicitation (α=.71) and child disclosure subscales (α=.76) demonstrated acceptable internal consistency.

Prior to the SSI, parents and teens each completed the Parental Knowledge questionnaire, which includes 5 items assessing the knowledge that parents have of their teen’s life [47,48]. Participants rate each item on a Likert scale from 1 (doesn’t know) to 5 (knows everything). Item scores were averaged to produce overall scores, with higher scores indicating greater parental knowledge. This measure exhibited strong reliability and validity, including across time, in a sample of young teens with type 1 diabetes [45]. In the present sample, the parent Parental Knowledge questionnaire demonstrated acceptable internal consistency (α=.63), and the teen Parental Knowledge questionnaire showed good internal consistency (α=.85).

#### Self-Efficacy for Parenting Adolescents Scale

Parent participants completed a modified version of the Self-Efficacy for Parenting Adolescents Scale (SEPA) before and immediately after the SSI. The original SEPA is a 23-item self-report measure of parents’ confidence in their ability to perform behaviors associated with effective parenting of teens [49]. Participants rate each item on an 11-point Likert scale from 0 (cannot do) to 100 (highly certain can do) in 10-point increments. For the purposes of this study, participants completed only 7 of the 23 original SEPA items. These 7 items were selected based on their relevance to the skills targeted by the SSI. The included items assessed parents’ confidence in their ability to respond appropriately, even when tired or irritable, when their adolescent is moody; be sensitive and nurturing when their adolescent has a negative attitude; spend positive, one-on-one time with their adolescent, without getting frustrated; be patient with their adolescent when in a hurry; engage in effective communication skills with their adolescent even when he or she is trying their patience; set reasonable limits for their adolescent (eg, rules and curfews); and do things to help their adolescent stay out of trouble at school. In addition to these 7 existing items, 3 new items were developed and added to assess skills targeted by the SSI but not captured in the original measure. These items assessed parents’ confidence in their ability to ask their teen every day about their plans for activities when unsupervised, follow up about their teen’s activities during unsupervised time, and work together with their teen to solve a problem. Mean scores were calculated for the overall modified scale and for individual items, with higher mean scores indicating greater parenting self-efficacy. In the present sample, the internal consistencies of the modified SEPA scale pre-SSI (α=.90) and post-SSI (α=.87) were good.

### Data Analysis

Descriptive statistics were used to characterize parent and teen SU and teen mental health, as well as to evaluate the acceptability and feasibility of the SSI. To assess acceptability, mean scores were calculated for the overall PFS and for individual items. To assess feasibility, the percentage of parent-teen dyads that completed the full SSI was calculated. Additionally, the percentage of parents and teens who selected each of the 3 family management skills was calculated. To assess the intervention’s potential usefulness, responses to family functioning surveys were examined to determine whether parents and teens selected areas of challenge with family management skills that aligned with the overall challenges they reported in these surveys. Additionally, paired-samples *t* tests were conducted to examine the difference in parenting self-efficacy (per SEPA) from pre- to post-SSI for both the overall scale and for individual items. Given the small sample size, each *t* test used a bootstrap procedure with 1000 resamples to calculate a 95% CI for the mean difference. All analyses were conducted using IBM SPSS Statistics (version 29.0.2.0; IBM Corp).

### Ethical Considerations

All study procedures were approved by the University of Vermont’s Institutional Review Board (institutional review board number 00002706), and all parents and teens provided informed consent and assent prior to their participation. Participant privacy was protected by assigning participant ID numbers and storing data in password-protected files on secure university servers. Parents and teens each received a US $75 Visa gift card for their participation in the study.

## Results

### Participants

A total of 38 families completed the initial interest form. Of these, 8 families passively declined screening by not responding to contact attempts, and 1 family was reached but declined to be screened. The remaining 29 families were screened by research staff, and 13 were found to be eligible. Of the eligible families, 10 scheduled and completed a consent call, and 9 of these 10 families scheduled and completed the SSI visit (Figure 1). At the study’s conclusion (December 2024), 3 eligible families were not consented yet due to both study team availability and having reached the study’s funding end date, but these families continued to express interest in participating in future studies.

Participants included young teens (n=9) and their parent (n=9). The average teen age was 13.11 (SD 0.78, range 12-14) years, and the average parent age was 49.22 (SD 5.65, range 41-56) years. The majority of teens identified as female (5/9, 55.56%) and White (7/9, 77.78%). All 9 parents identified as female and White. Of the 9 families, 4 (44.44%) had a mean annual family income below US $100,000 (range 31,000-330,000). Most parents (8/9, 88.89%) had completed college or an advanced degree (Table 1).

Among all 9 parent participants, 3 (33.33%) reported currently vaping tobacco, 4 (44.44%) reported never using cannabis, 2 (22.22%) reported past cannabis use, and 3 (33.33%) reported current cannabis use. Additionally, 1 parent did not report on history of problematic SU or SUD and 1 did not report on smoking tobacco. Among the reporting parent participants (n=8), a total of 3 (37.5%) parents reported a history of problematic SU or SUD, and 3 (37.5%) reported never smoking tobacco, 4 (50%) reported past tobacco smoking, and 1 (12.5%) reported current tobacco smoking.

Among teen participants (n=9), 4 (44.44%) had ever engaged in SU. The substances used included alcohol (3/9, 33.33% of teens), cigarettes (2/9, 22.22%), cannabis (1/8, 12.5%), and electronic vape (1/9, 11.11%). No teens reported trying other tobacco products (eg, chewing tobacco and cigars). Over half of the teens (5/9, 55.56%) screened positive for internalizing problems. Additionally, 44.44% (4/9) screened positive for attention problems and 11.11% (1/9) screened positive for externalizing problems.

**Figure 1:**
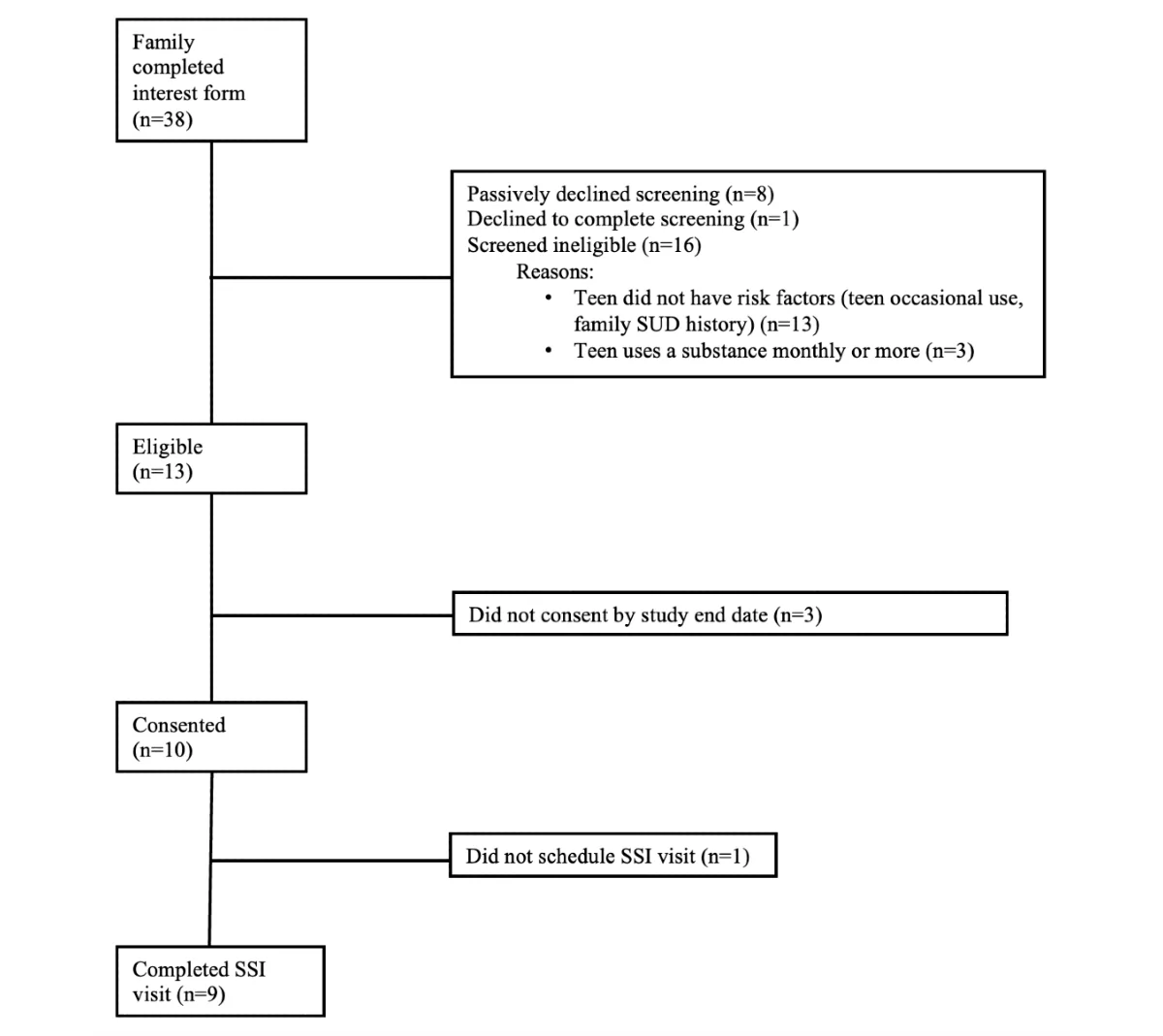
CONSORT (Consolidated Standards of Reporting Trials) diagram. SSI: single-session intervention; SUD: substance use disorder.

**Table 1:** Characteristics of parent participants (n=9) and teen participants (n=9).

Characteristics		Parents	Teens
**Sex, n (%)**			
	Female	9 (100)	5 (55.56)
	Male	0 (0)	4 (44.44)
**Race, n (%)**			
	Asian	0 (0)	1 (11.11)
	Black or African American	0 (0)	1 (11.11)
	White	9 (100)	7 (77.78)
**Highest educational level, n (%)**			
	Some college, no degree	1 (11.11)	—^a^
	Completed college	6 (66.67)	—^a^
	Completed advanced degree	2 (22.22)	—^a^

^a^Not available.

### Intervention Acceptability: Program Feedback Scale

All parents and teens who completed the SSI found it acceptable on all PFS items, as indicated by each single-item mean score and the overall mean score being ≥3 (Table 2).

**Table 2:** Means and standard deviations of Program Feedback Scale items among parents and teens completing the single-session intervention.

Items	Parents	Teens
I enjoyed the session	5.00 (0)	4.25 (0.71)
I understood what was taught in the session	4.89 (0.33)	4.63 (0.52)
I found the session easy to complete	4.78 (0.44)	4.13 (1.36)
I tried my hardest during the session	5.00 (0)	4.63 (0.52)
I found the session helpful	4.78 (0.67)	4.00 (0.53)
I would recommend the session to a friend	4.78 (0.44)	4.13 (1.13)
I agree with what I learned in the session	4.78 (0.67)	4.63 (0.52)
Overall score	4.86 (0.29)	4.34 (0.53)

### Intervention Feasibility: SSI Completion Rate

Of the 9 participating parent-teen dyads, 8 (88.89%) completed the full SSI, exceeding the feasibility threshold of 80% completion. In the 1 noncompleting dyad, the teen, with the family coach’s support, decided not to complete the action plan component of the SSI. This teen found it difficult to regulate their feelings about frustrations related to family communication; however, the parent did complete their action plan and the family coach helped them select goals related to addressing the teen’s expressed concerns about those communication challenges. This was the only deviation from fidelity to the intervention across all 9 dyads. Among the 8 fully completing dyads (n=16), all parents and teens successfully identified an area of challenge with family management skills to work on. The most frequently selected challenge area was Communication and Problem Solving (13/16, 81.25%), followed by Information Sharing (2/16, 12.50%) and Proactive Planning (1/16, 6.25%).

### Intervention Potential Usefulness

#### Congruence of Participant-Selected Area of Challenge With Self-Reported Pre-SSI Family Functioning

The area of challenge with family management skills most frequently selected by parents and teens in each dyad was improving Communication and Problem Solving. This choice was consistent with parent and teen reports on family functioning surveys (Table 3). While both parents and teens reported that parents had good knowledge of their teen’s life (per Parental Knowledge questionnaire), teens indicated that this knowledge was primarily attained through parental control, rather than through parental solicitation and teen disclosure (per PMSS). Additionally, teens reported lower quality communication with their parents (per IPPA), indicating that they tended to struggle to communicate their experiences to their parent.

**Table 3:** Family functioning metrics reported by parents and teens.

Measure			Parents		Teens
**IPPA^a^ Communication Subscale, mean (SD)**					
	Overall score	3.29 (0.47)		3.29 (0.71)	
**Parental Monitoring Short Scale, mean (SD)**					
	Parental control subscale	4.52 (0.80)		4.37 (0.73)	
	Parental solicitation subscale	4.22 (0.50)		3.30 (0.99)	
	Child disclosure subscale	3.70 (0.61)		3.74 (0.98)	
**Parental Knowledge Questionnaire, mean (SD)**					
	Overall score	4.25 (0.40)		4.07 (0.63)	

^a^IPPA: Inventory of Parent and Peer Attachment.

#### Self-Efficacy for Parenting Adolescents Scale

Following the SSI, parent participants reported greater overall parenting self-efficacy for skills taught in the SSI, with mean overall SEPA scores rising from pre-SSI (mean_pre_ 68.89, SD 15.83) to post-SSI (mean_post_ 81.78, SD 7.87). This increase was statistically significant with a large magnitude effect size (*t*_8_=3.38, 95% bootstrapped CI 6.22-20.44, bootstrapped *P*=.014, *d*=1.13).

At the item level, parents reported a statistically significant increase in confidence in their ability to be sensitive and nurturing when their adolescent has a negative attitude from pre- to post-SSI (mean_pre_ 61.11, SD 13.64; mean_post_ 73.33, SD 12.25; *t*_8_=4.40, 95% bootstrapped CI 6.67-17.78, bootstrapped *P*=.005, *d*=1.47). Parents also reported statistically significant increases in confidence in their ability to set reasonable limits for their adolescent (mean_pre_ 64.44, SD 29.20; mean_post_ 78.89, SD 16.16; *t*_8_=2.60, 95% bootstrapped CI 5.56-25.56, bootstrapped *P*=.034, *d*=0.87), in their ability to ask their teen every day about their plans for activities when unsupervised (mean_pre_ 72.22, SD 26.82; mean_post_ 93.33, SD 8.66; *t*_8_=2.80, 95% bootstrapped CI 7.78-36.67, bootstrapped *P*=.045, *d*=0.93), and in their ability to follow up about their teen’s activities during unsupervised time (mean_pre_ 67.78, SD 23.33; mean_post_ 91.11, SD 9.28; *t*_8_=3.40, 95% bootstrapped CI 11.11-35.56, bootstrapped *P*=.016, *d*=1.13). Differences in scores on all other individual items from pre- to post-SSI were not statistically significant, including items assessing parents’ confidence in their ability to respond appropriately, even when tired or irritable, when their adolescent is moody; spend positive, one-on-one time with their adolescent, without getting frustrated; be patient with their adolescent when in a hurry; engage in effective communication skills with their adolescent even when he or she is trying their patience; do things to help their adolescent stay out of trouble at school; and work together with their teen to solve a problem.

## Discussion

### Principal Findings

This study examined the initial acceptability and feasibility, as well as the potential usefulness, of a single-session family management skills intervention for young teens at high risk for SUD and their parent. Consistent with study hypotheses, findings indicated that (1) the SSI was acceptable to both parents and teens, (2) the SSI demonstrated adequate feasibility in terms of session completion, and (3) there was support for the SSI’s potential usefulness, as parents and teens selected areas of challenge with family management skills that aligned with the family challenges they reported, and (4) parents reported increased self-efficacy for those skills following the SSI.

Findings provided support for the SSI being both acceptable and feasible. All parents and teens found the SSI acceptable, as each single-item mean score and the overall mean score on the PFS were ≥3, meeting the a priori threshold for acceptability. On the PFS, parents and teens reported that they enjoyed the SSI, understood the content, found it easy to complete, tried their hardest during it, found it helpful, would recommend it to others, and agreed with what they learned. The SSI also demonstrated adequate feasibility, as of the 9 participating parent-teen dyads, 8 (88.89%) completed the full SSI, exceeding the a priori feasibility threshold of 80% completion. The high completion rate is notable, given that all teens in this study were considered high-risk (ie, they all engaged in occasional SU and/or had an immediate family member with an SUD history). Findings suggest that even among high-risk families, the SSI can be successfully implemented. However, the 1 teen who did not complete the action plan due to difficulty regulating emotions related to frustrations with family communication suggests that some families will still need a more traditional approach to care (eg, multiple sessions with a mental health professional). These findings are consistent with existing research on SSIs among youth with mental health symptoms that suggests this approach is highly feasible and acceptable to teens and families [22-24].

This study further supports the SSI’s feasibility by demonstrating its potential to be delivered by lay workers with no formal education in mental health and counseling. Specifically, the family coach for 7 of the 9 dyads in this study held a master’s degree in nutrition and food science and had no training as a mental health provider beyond the training they received in preparation for the coaching role. Existing research provides additional support for the use of lay workers in the delivery of mental health interventions. For example, a recent systematic review found that in the majority of randomized controlled trials conducted in low- and middle-income countries, interventions delivered by lay counselors without prior mental health training produced significantly greater improvement in mental health symptoms than usual care or no treatment [50]. Overall, findings from this study suggest that the SSI has the potential to be delivered effectively by lay workers, which could increase the accessibility of indicated prevention for young teens at high risk for SUD.

Moreover, findings provided support for the SSI’s potential usefulness. First, for this SSI to have usefulness for indicated prevention and remain highly scalable and accessible, the SSI must allow for individualized assessment and intervention, a hallmark of indicated prevention, without time-intensive intake procedures. To accomplish this, the SSI applies a Solution-Focused Brief Therapy framework that asks families to select the area of challenge in which they desire more support related to family management skills and offers intervention that is tailored to the individual’s goals and strengths. It was found that most parents and teens selected Communication and Problem-Solving as their top area for challenge for the SSI and that this was consistent with pre-SSI parent and teen ratings of family functioning, as the highest concerns for family functioning were reported in communication. This is consistent with existing research on SSI consultations that apply this framework [33] and studies examining Solution-Focused Brief Therapy [51-53], which support that patients can accurately identify top problems and generate helpful action plan steps based on strategies that have worked in the past for that patient.

Second, the potential usefulness of this SSI was supported by within person pre-to-post SSI increases in parenting self-efficacy on the family management skills targeted in the SSI. For example, parents reported significant overall increases in parenting self-efficacy, as well as increases in confidence in their ability to be sensitive and nurturing when their adolescent has a negative attitude, to set reasonable limits for their adolescent, to ask their teen every day about their plans for activities when unsupervised, and to follow up about their teen’s activities during unsupervised time. This is consistent with research suggesting that SSIs are effective for family behavior change [25]. In addition, the specific behaviors for which parents reported increases in confidence include those most important for SU prevention (ie, increased confidence in parental monitoring and limit setting, as well as increased confidence in responding to negative affect from their teen, an important skill for relationship building [54,55]). This suggests that the SSI is deserving of further assessment to determine whether it causes improvement in parenting self-efficacy.

### Limitations

The findings of this study should be considered in the context of its limitations. First, the study sample included only 9 parent-teen dyads, which reduced statistical power and limits the generalizability of the findings. Second, all parents in the sample identified as female and White, the majority of teens identified as White, and the mean annual family income was within the middle-class range; these factors may also limit the generalizability of the findings to a more diverse sample. Third, the study lacked a control group, so it is possible that the observed increases in parenting self-efficacy were due to factors other than the SSI. Fourth, the measure of parenting self-efficacy (ie, SEPA) was significantly adapted from its original validated format, with participants completing only a subset of the original items as well as unvalidated items that were added for the purposes of this study. However, the internal consistencies of the modified SEPA scale pre-SSI (α=.90) and post-SSI (α=.87) were good and comparable with the internal consistency of the original SEPA (α=.93) [49]. Additionally, scores on the modified SEPA scale pre-SSI were reasonably correlated with parent-reported scores on other measures of family functioning pre-SSI (0.37<*r*s<0.62). Fifth, participants completed the modified SEPA measure immediately following the SSI, so it is unclear whether the reported increases in parenting self-efficacy were maintained across longer periods of time. Finally, the backgrounds of the family coaches delivering the SSI were not consistent: 1 family coach had no training as a mental health provider, and the other coach had a master’s degree in clinical psychology. Thus, despite fidelity checklists suggesting strong fidelity to the intervention for both coaches, it is possible that there were inconsistencies in SSI delivery owing to the differing experience levels of the family coaches. Further work is therefore warranted to determine the ability of lay people to deliver the SSI.

### Future Directions

Future work should determine the SSI’s efficacy in improving parenting self-efficacy and preventing SUD through a large randomized clinical trial with a diverse sample and follow-up assessments. Furthermore, to triage care effectively, efforts should be made to identify which families are suited to the low-intensity SSI approach. Future work may also consider testing the SSI in older adolescents at high risk for SUD, as its approaches are also applicable for older-aged youth. Additionally, future research should investigate whether augmenting the SSI with a dyadic digital just-in-time adaptive intervention (JITAI) can promote family management skill use and behavior change beyond the SSI alone. A JITAI is defined as “an intervention design in which rapidly changing information about the individual’s internal state and context is used to decide whether and how to intervene in real time, in the individual’s natural environment” [56]. Specifically, JITAIs use mobile technologies to monitor an individual’s internal state and context and then provide support (eg, feedback and reminders) for healthy behavior to an individual at the exact time that support is needed [57]. Importantly, JITAIs have been shown to promote behavior change in both parents and teens [58]. A dyadic digital JITAI, which “address[es] the changing needs of two or more interconnected individuals” [56], could augment the SSI by targeting both the parent and the teen, leveraging and improving their social relationship to support behavior change. For example, a JITAI could send both parents and teens reminders to use family management skills, as well as offer shared access to digital features that promote daily connection and relationship building. Incorporating these ongoing supports into daily life could improve engagement with the family management skills and increase behavior change and SUD prevention across time.

Finally, the scalability of the SSI may depend on whether lay workers in schools and pediatric clinics are successful in delivering the intervention. Currently, limited access to family-based indicated prevention programs is in part due to challenges in developing the workforce for these programs that are led largely by licensed and trained mental health professionals [19]. Future work is needed to identify optimal strategies for training the existing lay workforce in schools and pediatric clinics to deliver this intervention. This research should be guided by a dissemination and implementation framework, such as the Consolidated Framework for Implementation Research [59], to determine which training strategies work in which contexts and why. A hybrid dissemination-effectiveness trial will also be essential to identify the best strategies for disseminating the SSI in schools and pediatric clinics, as well as its effectiveness in these real-world settings.

## Abbreviations

IPPA: Inventory of Parent and Peer Attachment
JITAI: just-in-time adaptive intervention
PFS: Program Feedback Scale
PMSS: Parental Monitoring Short Scale
PSC-17: 17-item Pediatric Symptom Checklist
S2BI: Screening to Brief Intervention
SEPA: Self-Efficacy for Parenting Adolescents Scale
SSI: single-session intervention
SU: substance use
SUD: substance use disorder

## Data Availability

The analysis code and materials used in this study are not openly available but are available upon request to the corresponding author. The raw data contained in this manuscript are not openly available due to privacy restrictions set forth by the institutional ethics board but can be obtained from the corresponding author following the completion of a privacy and fair use agreement.
